# Biomechanics of Disc Degeneration

**DOI:** 10.1155/2012/726210

**Published:** 2012-06-17

**Authors:** V. Palepu, M. Kodigudla, V. K. Goel

**Affiliations:** Departments of Bioengineering and Orthopaedic Surgery, Engineering Center for Orthopaedic Research Excellence (E-CORE), Colleges of Engineering and Medicine, University of Toledo, Toledo, 5046 NI, OH 43606, USA

## Abstract

Disc degeneration and associated disorders are among the most debated topics in the orthopedic literature over the past few decades. These may be attributed to interrelated mechanical, biochemical, and environmental factors. The treatment options vary from conservative approaches to surgery, depending on the severity of degeneration and response to conservative therapies. Spinal fusion is considered to be the “gold standard” in surgical methods till date. However, the association of adjacent level degeneration has led to the evolution of motion preservation technologies like spinal arthroplasty and posterior dynamic stabilization systems. These new technologies are aimed to address pain and preserve motion while maintaining a proper load sharing among various spinal elements. This paper provides an elaborative biomechanical review of the technologies aimed to address the disc degeneration and reiterates the point that biomechanical efficacy followed by long-term clinical success will allow these nonfusion technologies as alternatives to fusion, at least in certain patient population.

## 1. Introduction

Low back pain (LBP) remains the second most common symptom for a visit to a physician in the United States [1]. The associated costs may exceed $100 billion per year and are allied with lost wages and reduced productivity [2]. The pain may arise from any of the spinal structures (discs, facets, ligaments, vertebrae, and muscles), but one of the leading causes is spinal instability resulting from the degeneration of inter vertebral disc [3, 4]. Degenerative disc disease (DDD) encompasses disc herniation, spinal stenosis, and degenerative spondylolisthesis, among other changes. DDD becomes a source of chronic pain. Over 90% of spine surgeries are performed because of the DDD [5]. 

Intervertebral disc (IVD) is composed of nucleus pulposus in the central region surrounded by annulus fibrosis and cartilaginous end plates [6]. Nucleus is a hydrostatic fluid like structure, and it has a mixture of water and aggrecan-proteoglycan gel in combination with the collagen type II and elastin fibers network. Annulus, the other component of the disc, forms a structure of 15 to 25 “concentric” lamellae around the nucleus [6]. Each lamella is composed of collagen type I fibers, which is oriented at ±30° to the horizontal in consecutive layers. The IVD resists compression because of the osmotic properties of the proteoglycans [7]. Ability of the disc to resist anterior and lateral shears along with compression and flexion makes IVD the most important load bearing component of spine, beside the facets [8].

DDD is a part of aging, and it can occur due to many other factors as well. Mechanical factors like heavy lifting leading to abnormal loads, vibrations, immobilization, and trauma may implicate unfavorable distribution and transmission of stresses to adjacent spinal structures resulting in structural failures. Degeneration is attributed to structural failures such as annulus tears, disc prolapse, internal disc disruption, end-plate damage, and narrowed disc space [7, 9, 10]. Due to poor nutrition supply, water content of the nucleus decreases and the content of proteoglycans also changes (biochemical factors). This reduces the hydrostatic pressure and disc height altering the load distribution. The annulus and facet joints are overloaded to meet this demand. The annulus becomes “less” flexible in response to the increased compression, causing annulus fibers to tear. Annulus tears may cause a bulged or herniated disc, which further decreases the disc height. These may pinch the spinal cord or a nerve resulting in radiculopathy. The decreased disc height may trigger osteophyte formation across adjoining vertebrae and/or facet joint arthritis due to the increased loading on the neural arch by 40% [9]. End-plate damage that occurs decompresses the nucleus; the nucleus may protrude in to the vertebral bodies. The nucleus herniated through the end plate known Schmorl's node may cause inflammation [7]. Structural or mechanical damages also depend on loading history. These damages are irreversible in older population because of a decrease in the healing potential with age.

Although the degeneration of the disc takes place as a part of aging and other factors which are interrelated, the underlying mechanisms for the initiation of disc degeneration and its progression are still being pursued [5, 9].

## 2. Biomechanics of Disc Degeneration

A few of the pertinent biomechanical studies which emulate the mechanical factors that might affect the intervertebral disc and the concomitant spinal structures are presented in the following paragraphs.

Wilke et al. [11, 12] reported that the nucleus pulposus in the early life or in slightly degenerated discs acts like a gelatinous mass. A compressive load decreases disc height due to a decrease in the volume of gelatinous mass. This also increases the hydrostatic pressure which leads to a bulging of outer annulus. During the day, the compressive load reduces the disc height mainly because of water being squeezed out of the disc, and in part due to the creep of the viscoelastic annulus collagen fibers. Both effects are reversible in healthy discs like unloading of the spine during a night's bed rest [11]. The longer the load acts on spine, the more the annulus bulges and the more the facet joints are loaded. Disc degeneration alters the structure and function [13, 14]. Finite element (FE) studies showed that the risk of prolapse is highest in the posterior and posterolateral annulus, especially in normal and mildly degenerated discs, while moderate or strongly degenerated discs have a lower risk for a prolapse [15].

Prolonged sitting results in sustained axial compressive loading which may alter the viscoelastic properties of the disc and vertebra [16, 17]. Goel et al. found that an increase in load occurs across the disc at the resonant frequency of the spine 5 to 8 Hz range [18]. The resonant frequency can occur during driving and postures that are common in occupational workplace [19, 20]. This study found that at resonating frequency, the corresponding increase in nucleus pressure was about 150% of the static case, which implies that the spine would be exposed to excessive loads.

Kong et al. conducted an FE study in which muscle dysfunction due to quasistatic backlifting conditions was simulated and found that muscle dysfunction destabilized the spine, reduced the role of facet joints in transmitting load, and shifted loads to the discs and ligaments [21].

Wang et al. [22] conducted a study on ten symptomatic patients with DDD and reported that the discs at the adjacent levels experienced higher tensile and shear deformations during end ranges of lumbar motion, compared to the healthy subjects. The authors also evaluated the effect of lumbar DDD on *in vivo* motion of the facet joints under functional weight bearing activities and concluded that the DDD alters the facet joint motion at the degenerated and adjacent levels. They also observed the hypermobility in coupled rotations implying a biomechanical mechanism leading to further adjacent level degeneration [23].

Disc degeneration at one or multiple levels may affect the other spinal component of that level or other levels [3, 24]. Panjabi et al. [24] found that any damage to disc alters the biomechanics of facet joints by disproportionately sharing the facet loads.

The relationship between the intervertebral disc degeneration and nonlinear multidirectional spinal flexibility was investigated by Mimura et al. [25]. They studied 47 lumbar discs under sagittal, frontal, and transverse plane loadings (pure moments) and found that the range of motion (ROM) decreased in flexion-extension and lateral bending. The neutral zone-to-range of motion (NZ/ROM) ratio increased for all the three rotations, indicating greater joint laxity with degeneration.

Another study measured the stress distribution *in vitro* in normal, healthy discs and degenerated discs under compression [26]. They found that the stress distribution was uniform, isotropic for normal disc; nonuniform and anisotropic for the degenerated disc.

Shirazi-Adl et al. [27] performed a FE study in which they simulated a characteristic of the degenerated disc, that is, 50% loss of disc pressure than that of normal disc and subjected the motion segment to sagittal plane pure moments up to a maximum of 60 Nm. They found that a 50% reduction in intradiscal pressure had a decrease in the segmental stiffness. Additionally, they reported that the flexion rotation had lower intradiscal pressure in a disc with pressure less than in a normal disc, indicating that the portion of load transmitted through the nucleus decreases with degeneration.

**Figure 1:**
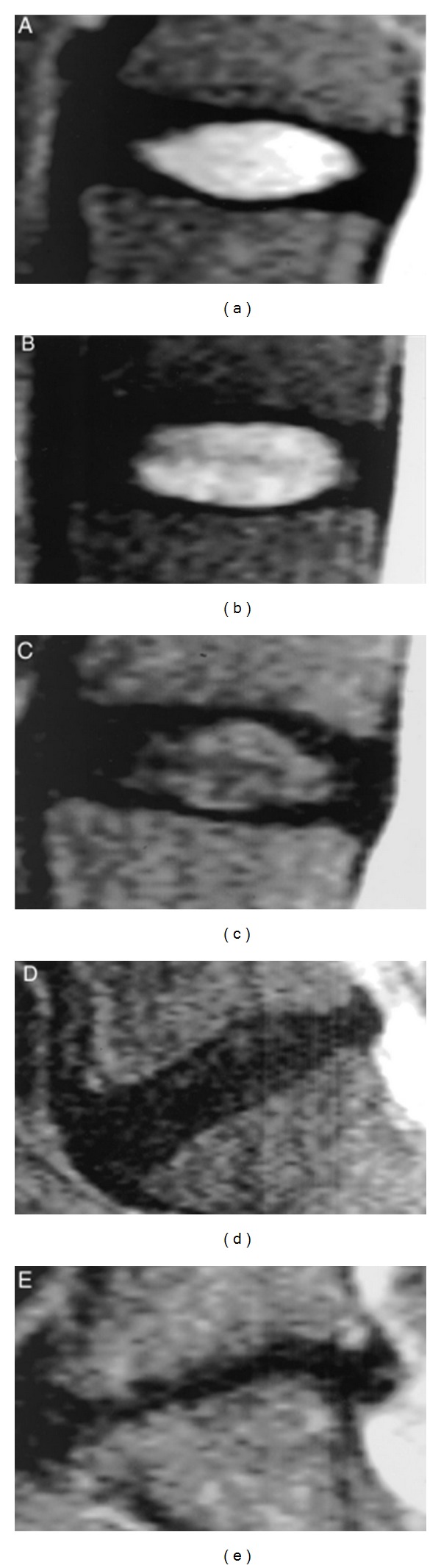
(a–e) pictures depict the grading system for the assessment of lumbar disc degeneration. Grade I: the structure of the disc is homogeneous, with bright hyperintense white signal intensity and a normal disc height. Grade II: the structure of the disc is inhomogeneous, with a hyper intense white signal. The distinction between nucleus and annulus is clear, and the disc height is normal, with or without horizontal gray bands. Grade III: the structure of the disc is inhomogeneous, with intermediate gray signal intensity. The distinction between nucleus and annulus is unclear, and the disc height is normal or slightly decreased. Grade IV: the structure of the disc is inhomogeneous, with hypointense dark gray signal intensity. The distinction between nucleus and annulus is lost, and the disc height is normal or moderately decreased. Grade V: the structure of the disc is inhomogeneous, with hypo intense black signal intensity. The distinction between nucleus and annulus is lost, and the disc space is collapsed. Grading is performed on T2-weighted midsagittal (repetition time 5000 msec/echo time 130 msec) fast spin-echo images [28].

Some authors have devised ways of grading the level of disc degeneration (Figure 1; Table 1) in the lumbar spine based on the MRI images [28].

The level of disc degeneration varies among patients and so does the type of treatment. The treatment may range from conservative treatment such as bed rest and prescription of pain relievers for mild disc degeneration to surgical intervention in severe chronic degeneration cases.

It is essential to treat DDD to relieve pain and possibly prevent further degeneration at index level and adjacent levels. Conservative treatment includes chiropractic adjustments, physical therapy, yoga, acupuncture, and medication. If conservative treatment fails, surgery would be the next option. Spine surgery involves different surgical techniques, using appropriate instrumentation to relieve pain. There are many surgical treatments such as fusion with or without rigid instrumentation and nonfusion techniques like dynamic stabilization, total disc arthroplasty, and implanting interspinous devices [3].

## 3. Biomechanics of Spinal Implants

Biomechanics of the spine is altered by the implantation of spinal devices used to stabilize the segment [29]. Along with many devices currently available in the market to treat spinal disorders, many new designs are also being developed in the hope to improve clinical outcomes. It is essential to evaluate their biomechanical efficacy among other issues, prior to clinical use [30]. The spinal implants can be evaluated by comparing the stability of the construct to the intact spine stability and/or stability provided by a predicate device. The biomechanical effects of decompression and stabilization provided by implants can be assessed using *in vitro *studies [31, 32]. *In vitro* studies involving ligamentous spine specimens from human cadaver or other species like sheep, calf, and rabbit are carried out using standard test protocols [30]. Finite element analysis (FEA) in spine biomechanics is very helpful to perform the structural analysis of bone and bone implant composites of complicated geometry. Since it is difficult to get all the parameters from experimental studies, finite element models can be used to address the remaining issues [33, 34]. Thus, *in vitro* and FE-based biomechanical studies provide valuable information on implants safety and effectiveness prior to their clinical use [30].

### 3.1. Fusion Systems

Fusion restricts the motion of involved segment. It may reduce progressive degeneration and relieve the patient from back pain. The main clinical indications for fusion are failed conservative treatment, prolonged back pain more than a year, and advanced degenerated disc [3]. Fusion surgeries are performed with or without supplement instrumentation. Segment fusion is achieved through the use of autograft, allograft, bone graft substitute, demineralized bone matrix (DBM), ceramic-based bone graft, recombinant human bone morphogenetic proteins (rhBMP-2), *β*-tricalcium phosphate (TCP), calcium sulphate (CaS), and hydroxyapatite (HA) [3, 35]. Fusion has been the gold standard in treating DDD and practiced since the beginning of the 20th century. Fusion without instrumentation has often led to nonunion of bone known as pseudoarthrosis. To overcome this complication, many spinal implants have been developed which are now used in fusion surgeries. The usage of spinal instrumentation provides segmental stability and facilitates high fusion rates.

**Figure 2:**
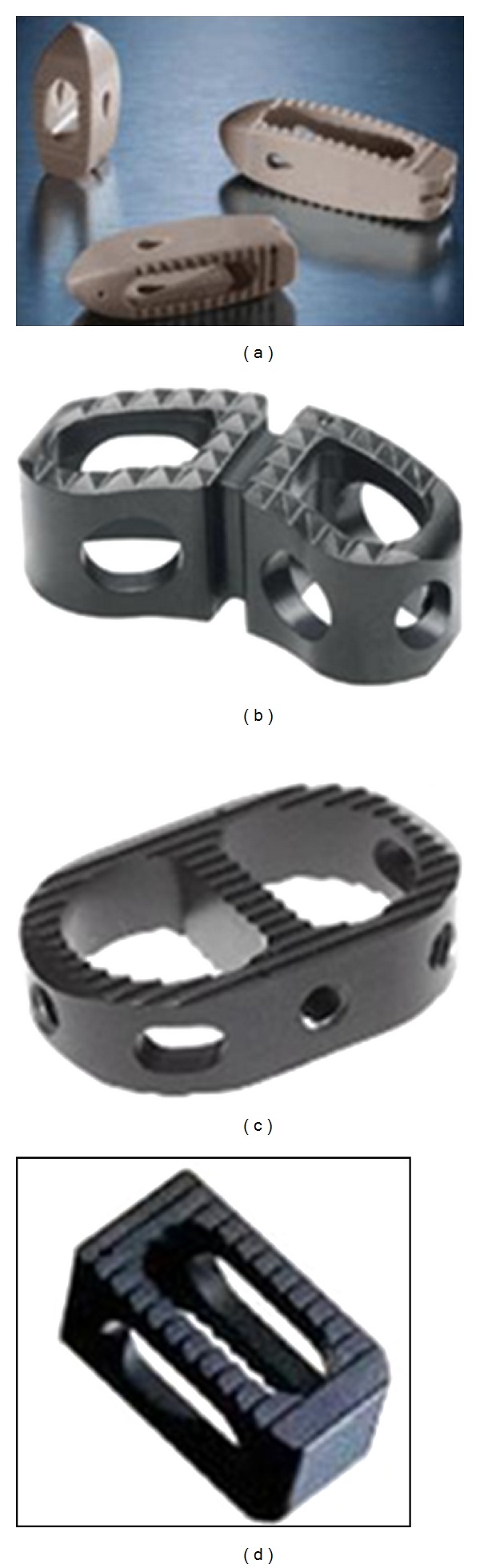
Rigid interbody cages. (a) Ardis (Zimmer spine, Minneapolis, MN, USA), (b) Leopard (DePuy, Raynham, MA, USA), (c) Cougar (DePuy, Raynham, MA, USA), and (d) Jaguar (DePuy, Raynham, MA, USA) (website).

**Figure 3:**
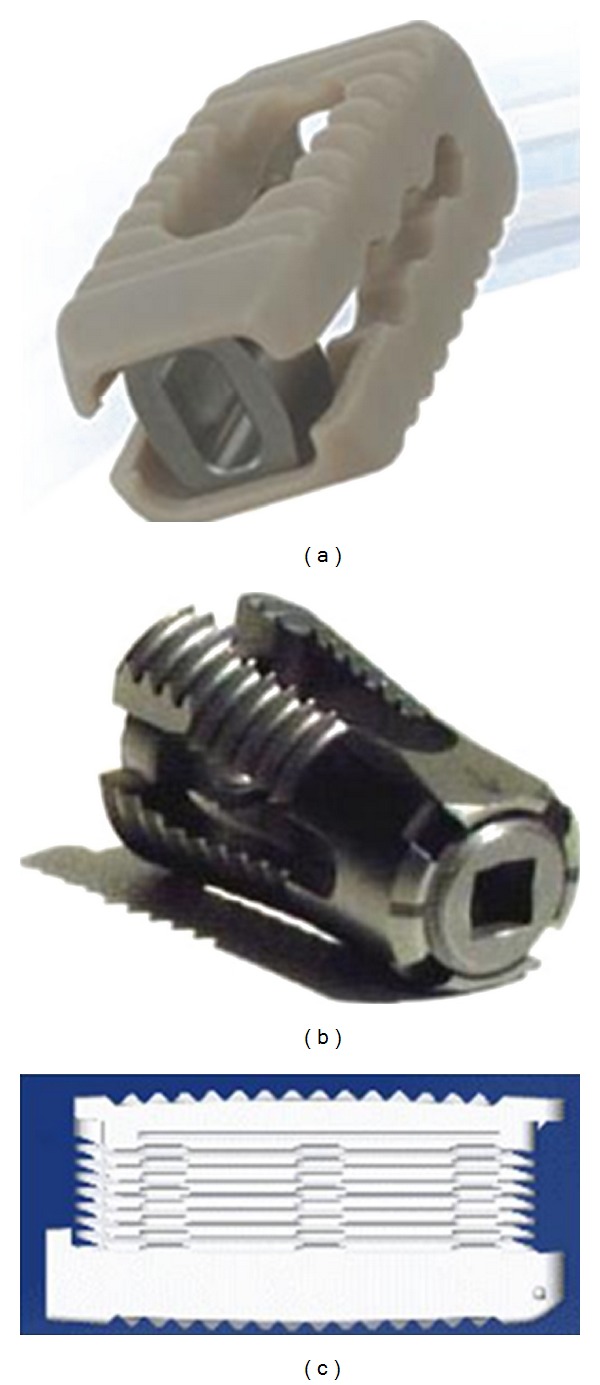
Expandable interbody cages. (a) Varian (Medyssey spine, Skokie, IL, USA), (b) VariLift-L (Wenzel spine, Austin, TX, USA), and (c) StaXx XDL (Spine wave, Shelton, CT, USA) (website).

Lumbar interbody fusion (LIF) was introduced by Cloward, and currently, it is being used widely [3, 36]. In LIF, cages filled with bone graft are placed in the disc space, supported by instrumentation to stabilize the spine and thereby enhance the fusion process. The bone grafts placed in between the vertebrae experience 80% of compressive loads, which enhances the fusion process. The grafts in LIF occupy 90% of bony area in between the vertebrae, which has rich vascular supply leading to enhanced fusion [36]. The cages were initially designed as rigid systems (Figure 2) in cylindrical, rectangular, and other shapes. However, to overcome the problems associated with these rigid cages [37], expandable cages (Figure 3) have been developed in recent times.

**Figure 4:**
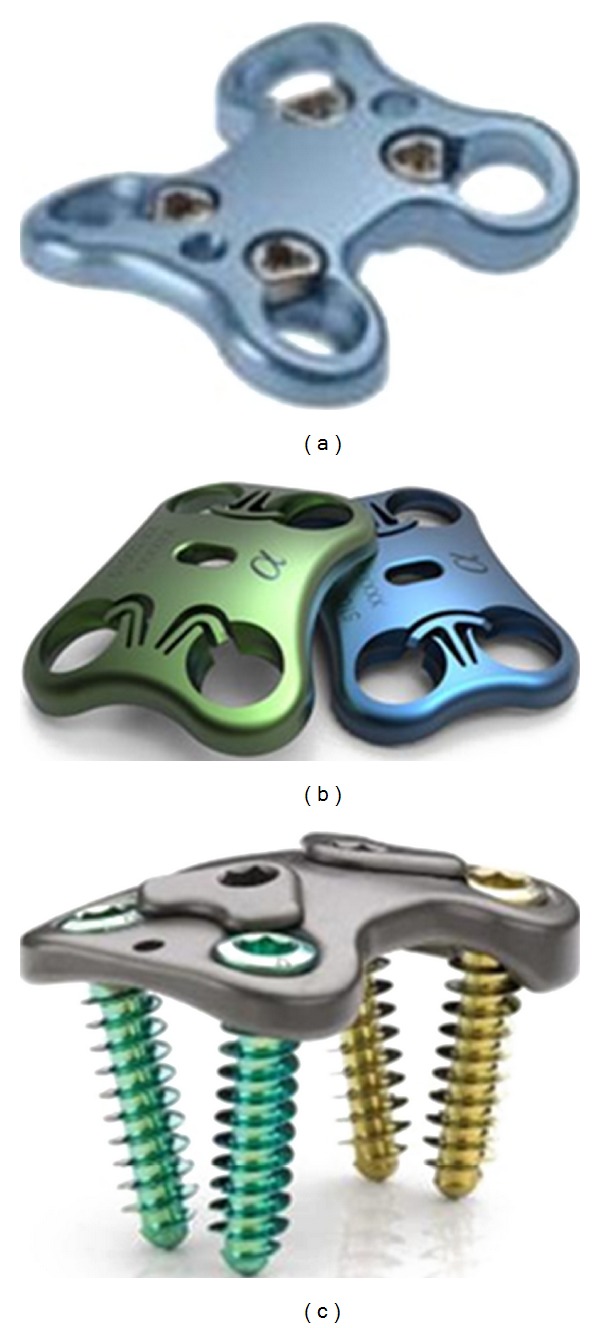
Anterior plate system. (a) Aegis (DePuy, Raynham, MA, USA), (b) Aspida (Alphatec spine, Carlsbad, CA, USA), and (c) Trinica (Zimmer spine, Minneapolis, MN, USA) (website).

**Figure 5:**
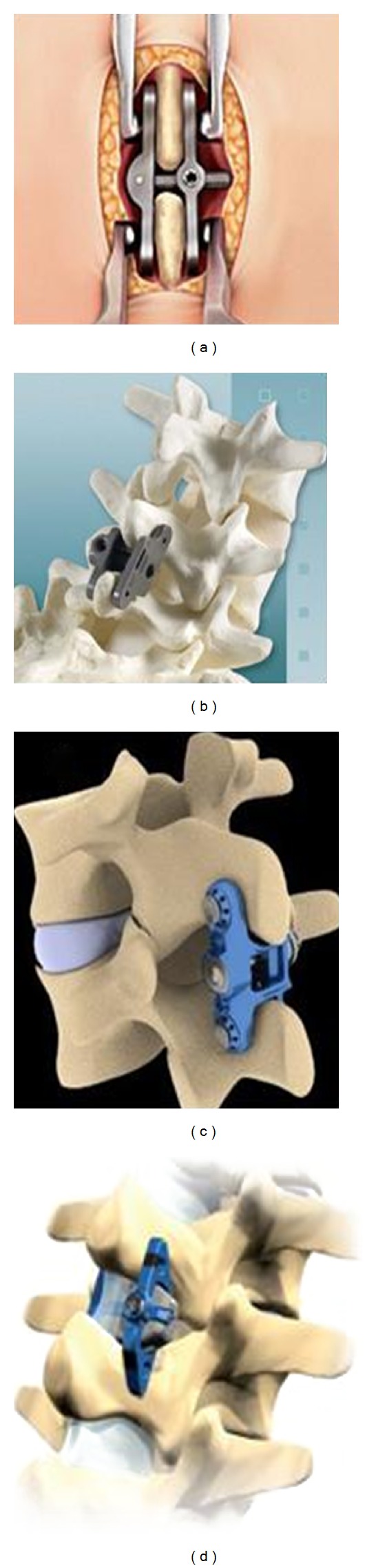
Interspinous fusion devices. (a) CD Horizon spire (Medtronic, Memphis, TN, USA), (b) Aspen (Lanx, Inc., Broomfield, CO, USA), (c) Prima LOK (OsteoMed, Addison, TX, USA), and (d) Axle (X-Spine, Miamisburg, OH, USA) are currently being studied (website).

There are several implants used as spinal instrumentation in fusion procedures like pedicle screw system and rods, plates (Figure 4), clamps, and wires. Pedicle screw system is considered to be an effective supportive instrumentation in achieving highest fusion rates [38]. Interspinous fixation systems (Figure 5) are also currently being developed and are gaining some popularity as their performance is similar to standard pedicle screw system [39, 40]. Interspinous devices are implanted by minimally invasive procedures in the posterior region, and they are also used in conjunction with interbody fusion procedures.

There are both anterior and posterior approaches for fusion surgery. The posterior approaches include posterolateral fusion (PLF), posterior lumbar interbody fusion (PLIF), and transforaminal lumbar interbody fusion (TLIF). Anterior approach includes anterior lumbar interbody fusion (ALIF) and extreme lateral inter body fusion (XLIF). XLIF, which is gaining popularity recently, involves lateral accessing of anterior column using sophisticated imaging technology to avoid neural disruption [41]. This procedure has the advantage of overcoming the complications associated with PLF, PLIF, TLIF, and ALIF. Combination of both anterior and posterior approach is called anteroposterior fusion, also known as 360° fusion. Depending on the level of surgery, sex of the patient, anatomic variations, and history of spine surgery, one or combination, of the aforementioned procedures is selected to treat LBP, and it is surgeon-specific [42].

A biomechanical study was performed by Kiapour et al. [43] using finite element (FE) technique to evaluate the effect of VariLift expandable and BAK cages on biomechanics of the lumbar spine motion segment. The cages were simulated at the L4-L5 level using PLIF surgical approach. The VariLift cage depicted comparable biomechanical effects on the lumbar segment with those of BAK cage. The expansion mechanism led to a relatively larger contact area between the cage and the endplate improving the chances of solid fusion to occur after surgery. The expansion of the cage also follows the lordotic angle of the treated segment ensuring a better contact between the cage and endplates.

**Figure 6:**
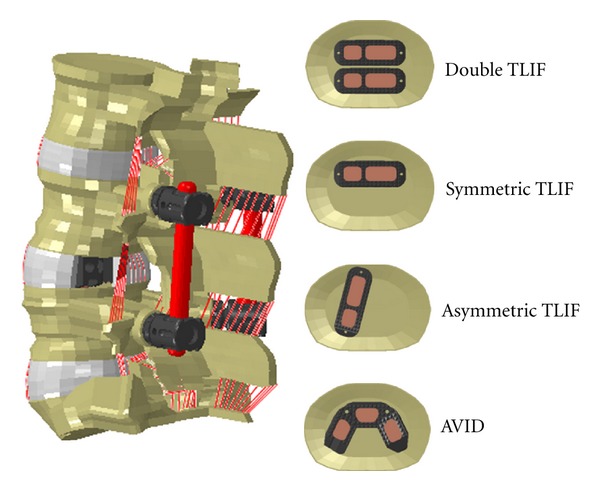
Four configurations of the interbody devices implanted at L4-L5 level in the FE model. (a) double cage TLIF; (b) regular TLIF Symmetrically placed; (c) regular TLIF asymmetrically placed; (d) large footprint TLIF (AVID) [44].

The footprint size of the interbody fusion device is an important factor that determines the biomechanical stability afforded by these implants. Moreover, occurrence of subsidence is also influenced by the cage's footprint. A finite element (FE) analysis was conducted by the same group [44] to compare the loading and stresses at vertebral endplates following implantation with AVID TLIF cage of a larger foot print compared to regular TLIF cage in different configurations (Figure 6). A follower load of 400 N was applied to the spine to simulate compression (at standing posture), and then, a 10 Nm bending moment was applied to the segment to simulate physiological flexion and extension loadings. They found that the double TLIF and AVID cases observed slightly higher normal loads at the endplates compared to other cases in all loading modes due to their higher contact area at the interface. The larger footprint interbody device (AVID) resulted in lower stresses in the endplate immediately after surgery. AVID implant may be able to lower the incidence of subsidence, as compared to regular TLIF devices.

Oxland et al. [45] and Rathonyi et al. [46] conducted cadaver biomechanical studies in which they evaluated anterior lumbar interbody fusion (ALIF) cages and observed a decrease in stability in extension. In flexion, lateral bending, and axial rotation, the stabilization was significant compared with the intact spine (the median value for motion was 40, 48, and 29 percent of the value for the intact condition, resp.; *P* = 0.002  for all three directions). In this study, stabilization was defined as a decrease in motion after insertion of an implant.

Tsantrizos et al. performed a cadaver study with Ray TFC and contact cages using posterior approach (PLIF) and reported that the stability in axial rotation decreased significantly, more with Ray TFC than with the other cages [47].

Another study performed by the Kiapour et al. [48] simulated the cadaveric experiment of Kanayama et al. [49] using FE technique. The load-displacement behavior and stresses in compression (500 N), flexion (5 Nm), left bending (3 Nm), and left rotation (50 N + 3 Nm) were computed for 4-WEB cage followed by comparison with two titanium (BAK and TITAN) cages and one PEEK interbody cage. The maximum pressure on the bone graft was 123.5, 304.5, 58.6, and 145.8 KPa in the WEB cage with smaller foot print (comparable to other cages) compared to 113.7, 144.1, 64.1 and 121 KPa in PEEK, 146.4, 132.6, 57.5, and 160 KPa in TITAN, and 30.7, 82.7, 17.7, and 36 KPa in the BAK device. The 4-WEB implanted segment had lesser peak stress at the interface with bony endplates.

The XLIF surgical procedure was simulated in a FE study by Kiapour et al. [50] on lumbar-pelvis segment to compare the biomechanics of interspinous fixation device with traditional screw-rod fixation system. Segmental motion and loads on sacroiliac joint (SIJ) and vertebral endplates were computed for all cases after applying a 400 N of compressive load and 10 Nm moment. They reported that the placement of fixation constructs leads to a significant decrease in range of motion of all index levels (L2–L5) in all loadings. At each of implanted levels the motion decreased by about 95% (Flex), 93% (Ext), 80% (LB), and 90% (LR) in interspinous device implanted model compared to intact case. The reductions in motion were 97%, 95%, 96%, and 94% for screw fixation and 51%, 48%, 68%, and 86% for cage alone cases, for same loadings, respectively. Also, the maximum load at SIJ decreased by 4% in Flex and increased by 8% in Ext, 8% in LB, and 7% in LR for all implanted cases compared to intact case. In the posterior plate model, the shear load at endplates of the most superior implanted segment increased and decreased in extension and left bending loadings, respectively compared to other fixation constructs.

Both cadaver and FE studies evaluated standalone cages and reported less stabilization of the spine in the literature. Anterior or posterior instrumentation systems along with cages are essential to have proper stability at the implanted level. However, expandable cages may provide enough stability without additional instrumentation [43].

The main complication associated with fusion is adjacent segment degeneration. The reason for this has not yet been clear and has become the point of debate. Some people argue that degeneration at adjacent level is part of aging spine, and others argue that it is due to reduced motion resulted from fusion. The reasons for adjacent level degeneration can be hyper mobility, increased disc pressure, increased facet joint pressure, and alteration in histological properties of ligaments at adjacent level to the index level [51]. Many *in vitro* and FEA studies showed the adjacent level hyper mobility after fusion [34, 52–56], but there are very few *in vivo* studies [51], which showed that the adjacent hyper mobility was not significant.

The success of the fusion surgery is defined as achieving arthrodesis across index level to provide stability and relieve pain. The modern techniques are successful in achieving fusion in 95% of the cases; however, the pain in the low back is relieved in less than 70% of the cases [52]. In spite of its wide application, fusion has varied clinical outcomes [3, 52, 53]. The causes of adjacent segment degeneration were not clear, though they are attributed to reduced motion at index level and increased motion at the adjacent level. In order to overcome morbidity associated with fusion, motion preservation devices were developed [52, 57].

The literature review of fusion systems enumerates major drawbacks like restricted (or) lack of motion, pseudoarthrosis, adjacent level degeneration, and donor site pain. The above shortcomings of fusion have led the researchers to develop an alternative approach for the treatment of disc degenerative disease.

Many non-fusion techniques have been investigated and have emerged in recent times to replace the conventional fusion techniques in treating degenerative discogenic pain. These techniques include spinal arthroplasty (artificial disc and nucleus) and dynamic stabilization systems. These systems aim to provide a more physiologic solution.

### 3.2. Total Disc Replacement

Disc arthroplasty or total disc replacement is one such option that is being seen as a potential alternative to fusion. As the name suggests, the goal of disc arthroplasty is to completely replace the degenerated intervertebral disc by an artificial implant which has capability not only to treat the pain causing symptoms but also promises to restore the lumbar motion and create a proper load balance with surrounding tissue without compromising patient safety.

**Figure 7:**
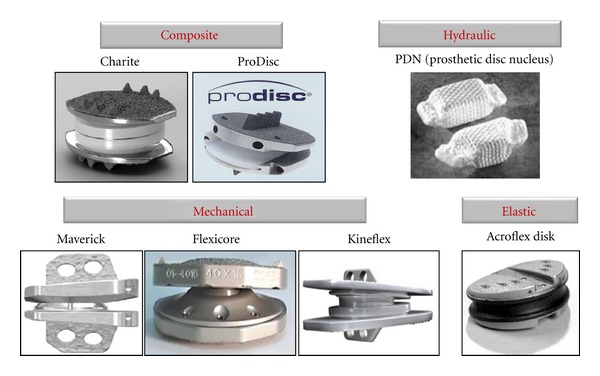
Different lumbar artificial disc concepts: Composite (Charité, Prodisc), Hydraulic (PDN), Mechanical (Maverick, Flexicore, Kineflex), and Elastic (Acroflex) [60].

The first human implantation of lumbar artificial disc was performed by Fernstrom in 1966 [58]. He used a metal ball (SKF ball bearing) to reproduce the mechanism of the disc. However, the obtained results were poor, and the implant was withdrawn. The SB Charité prosthesis, the first FDA-approved artificial disc for clinical use in USA, was designed in the former East Germany in the early 1980s by Schellnac and Buttner and was first implanted by Zippel in 1986 [59]. This event triggered the development of several variety of artificial discs aiming on parameters like restoring natural motion, biocompatibility, corrosion and wear resistance, stability, strength to sustain maximum expected loads, maintain intervertebral height, preserve lordosis, and to restore the energy absorptive qualities of the native disc.  Table 2 lists and Figure 7 depicts some of the major lumbar artificial disc designs. The present lumbar disc designs can be classified into four groups:

- composite discs: comprise of several articulating parts; often with different materials (Charité, andProDisc);
- hydraulic discs: these are designed for nucleus replacement and include an expandable fluid enclosed by a woven/porous bag (PDN);
- mechanical discs: which are made of articulating parts made of single type of material (Maverick, Flexicore, and Kineflex);
- elastic discs: include a deformable cores, usually made of elastomers or polymers attached to metallic endplates (Acroflex).

These artificial discs are also classified based on constraint parameter as constrained, semiconstrained, and unconstrained, respectively. The unconstrained design strategy allows for six-degrees-of-freedom segmental motion, with translations and rotations about three independent axes. Constrained devices typically permit rotation in all planes and include a fixed center of rotation, which limits segmental translation under flexion-extension and lateral bending conditions [61].

Biomechanical data from *in vitro* and mathematical modeling are presented. Different biomechanical parameters such as segmental motion, instantaneous axis of rotation, intradiscal pressure, facet loads, load/stress distribution at bone-implant interface, and wear at articulating sites, have been analyzed after disc replacements to understand device's ability to mimic the intact disc behavior and predict its durability in the long run.

#### 3.2.1. *In-Vitro* Studies

The *in vitro* studies enable us to understand the effects of total disc arthroplasty (TDA) on the kinematics of the implanted and adjacent levels of the spine. 

Hitchon et al. studied the biomechanics of Maverick anterior disc using an *in vitro* setup with 7 human lumbar specimens, in which pure moments of 6 Nm were applied in all planes of rotation after implanting the artificial disc at L4-L5 level [64]. They observed that the artificial disc decreased flexibility compared to discectomy, and the motion was comparable with the intact state.

Rousseau et al. did an *in vitro* study on twelve human lumbar spine segments after disc replacement with Prodisc II (6) and Charité III (6) versus intact. They measured the facet forces and instantaneous axes of rotation (IAR) for different spinal positions under simulated weight-bearing conditions. They concluded that the degree of constraint affects postimplantation kinematics and load transfer. With the Prodisc (3 DOF), the facets were partially unloaded, though IAR did not match the fixed geometrical center of the UHMWPE. The latter observation suggests joint surface incongruence is developed during movement. With the Charité disc (5 DOF), the IAR was less variable, yet the facet forces tended to increase, particularly during lateral bending. These results highlight the important role the facets play in guiding movement, and that implant constraint influences facet and implant synergy [65].

Ha et al. [62] conducted a study on five L2-S2 spines in which range of motion, facet strains and intradiscal pressures were monitored. A 400 N compressive load and 8 Nm moments in all three planes were applied to compare the intact, postimplantation of Semiconstrained Activ-L device at L4-L5 level. They reported that even though the device could not restore the normal motion of the intact spine, results of other parameters implicated a reduction in the incidence of adjacent segment disease. Those parameters were insignificant decrease of intradiscal pressure at the inferior adjacent disc, and the statistically significant decrease of facet strains at the operative level during flexion and strains at the inferior facets in axial rotation.

Goel et al. studied the biomechanics of spine implanted with Charité disc using a hybrid loading protocol [66]. They employed both *in vitro* experiment and finite element modeling. Results indicated that the Charité artificial disc placement slightly increased motion at the implanted level, with a resultant increase in facet loading when compared to the adjacent segments. The motions and loads were less at the adjacent levels.

Most of the lumbar artificial discs are of articulating type. These have potential for wear, much like the hip and knee arthroplasties. Cyclic loading and relative motion at the bearing surface may increase the risk to surrounding spinal structures like spinal cord and blood vessels. Therefore, biotribological tests serve as an effective preclinical tool to investigate device wear characteristics. A wear rate of 1.1 mg/million cycles [67] has been reported for the Charité artificial disc. Paré et al. [68] reported a steady state wear rate of 0.33 ± 0.12 mm^3^/million cycles in flexion-extension and 0.43 ± 0.06 mm^3^/million cycles in combined motion tests for the metal-on-metal Maverick disc (constrained).

#### 3.2.2. FE Analyses

A finite element study was conducted by Rohlmann et al. to understand the effects of ProDisc on lumbar spine kinematics. They loaded their model with the upper body weight and muscle forces to simulate standing, 30-degree flexion, 15-degree extension, and 6-degree axial rotation. The disc position was varied by up to 2 mm in both the anterior and posterior direction. Three different disc heights were investigated as well as the influence of removing different portions of the natural disc and resuturing the ALL ligaments. They observed that implant position strongly influenced intersegmental rotation for the loading cases of standing and flexion. Also, they found that a disc height 2 mm in excess of the normal disc space increased intersegmental rotation at implanted level during standing and extension. The intersegmental rotations were closer to the intact spine, when lateral portions of the annulus were not removed. Finally they concluded that when implanting an artificial disc, great care should be taken in choosing the optimal height and correct position for the implant. Lateral portions of the annulus should be preserved whenever possible. A perfect reconstruction of the ALL would help restore the biomechanics to normal [69].

Moumene and Geisler [70] performed a study to evaluate the loading on the facet joints and stress on the polyethylene core after implantation of Charité (unconstrained) and Prodisc (Semiconstrained) TDA. The unconstrained TDA unloads the facet joints and presents decreased core stress as compared to the fixed-core Semiconstrained TDA.

In a computational study performed by Dooris et al. [71], the effects of facet load sharing following TDA were examined. Different annular window sizes and varied antero-posterior artificial disc placement was simulated for a ball-on-socket disc design by Medtronic. Findings demonstrated that an artificial disc can alter spinal bending stiffness in the sagittal plane. Changes in spinal stiffness were noted to be dependent on the position of the disc and degree of annular resection. Anterior placement of the device led to increased facet joint loads in compression and extension. These findings suggest that if the anterior longitudinal ligament is preserved and the implant is placed posteriorly within the disc, the spinal stiffness will be restored, and facet loads will be maintained at preimplantation levels.

A study performed by Denozière and Ku [72] to compare TDA and fusion at one level of lumbar spine indicated that the level implanted with the artificial disc showed excessive ligament tensions (greater than 500 N), high facet pressures (greater than 3 MPa), and a higher risk of instability. The mobility and the stresses in the level adjacent to the arthroplasty also increased. They concluded that there was a greater risk of instability and further degeneration for artificial disc implanted model than that predicted for the fused model.

**Figure 8:**
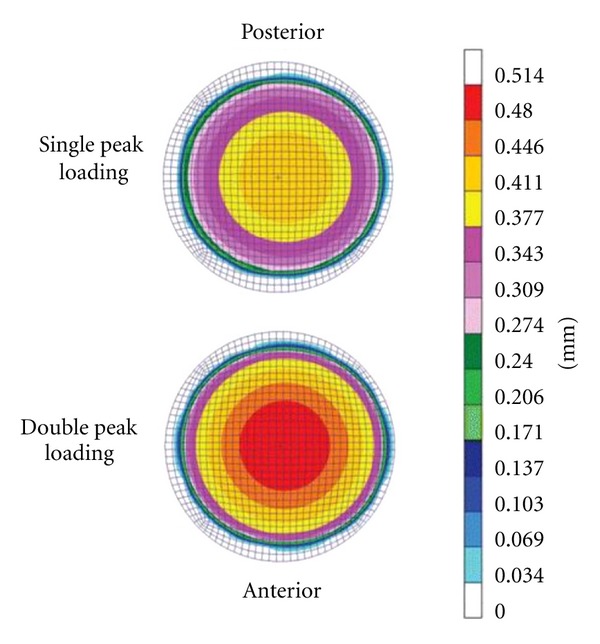
Linear wear contour predicted for ProDisc-L using finite element technique. Adapted from [62].

FE models have also been utilized to understand wear characteristics of joint replacements in the hip and knee. FE-based wear study was conducted by Rawlinson et al. [73], which depicted a uniformly distributed wear pattern as per ISO 18192 which was not observed during the retrieval analysis (Figure 8). This study was validated against experimental wear simulation of ProDisc-L implant.

FE technique was also applied in cervical spine by Bhattacharya et al. [74] to evaluate wear in a simulated C5-C6 FSU. A predictive FE wear model of the artificial disc alone (TDR only) was developed, and it was implanted into C5-C6 FE model (TDR + FSU). Both of these models were subjected to a motion profile (rotation about three axes) with varying preloads of 50 to150 N at 1 Hz, consistent with ISO 18192. A subroutine based on Archard law simulated abrasive wear on the polymeric core up to 10 million cycles. The TDR + FSU model was further modified to simulate facetectomy, sequential addition of ligaments, and compressive load. They reported more predicted localized wear in certain regions for TDR + FSU, in contrast to the uniformly distributed wear pattern of the TDR-only model. In addition, the cumulative volumetric wear for the TDR-only model was 10 times that of the TDR + FSU model. The TDR + FSU model also revealed a separation at the articulating interface during extension and lateral bending. After facetectomy, the wear pattern remained lopsided, but linear wear increased eight-fold, whereas volumetric wear almost tripled. This was accompanied by a reduction in observed liftoff.

Similar kind of studies in the lumbar spine may enable the scientists to pursue and understand the effects of clinical and other parameters (like surgical variables, different loading profiles, different disc designs, and bone quality) on wear of lumbar artificial discs. 

#### 3.2.3. *In Vivo * Studies

In the *in vivo* study of Siepe et al. [75], 175 patients with disc replacement with mean followup of 29.3 months were investigated. Facet joint pain, predominantly at the index level, was identified in 22 patients (12.6%). The sacroiliac joint was also a frequent cause of post-operative pain (*n* = 21; 12.0%). Pain from both structures influenced all outcome parameters negatively (*P* < 0.05). Patients with an early onset of pain ≤ 6 months) were 2–5 fold higher at risk of developing persisting complaints and unsatisfactory outcome at later stages in comparison to the entire study cohort (*P* < 0.05). They also observed that the level of TDR significantly influenced postoperative outcome. Best results were achieved for the TDRs at L4/5 (incidence of posterior joint pain: 14.8%). Inferior outcome and a significantly higher incidence of posterior joint pain were observed for TDR at L5/S1 (21.6%) and bisegmental TDR at L4/5/S1 (33.3%), respectively. Their study was unable to address that TDR will reduce the incidence of posterior joint pain, unlike the lumbar fusion procedures.

Zigler [76] did a clinical study on 78 patients with minimum 6-month followup replacement of ProDisc. Among the patients, 54 also had a 1-year followup, enrolled in a prospective randomized FDA study evaluating the safety and efficacy of ProDisc II versus control, a 360-degree lumbar spinal fusion. At 6-month follow-up, there were 55 ProDisc patients out of which 23 underwent fusion. Both fusion and disc replacement group had similar clinical outcomes. Also a trend was identified at 6 months in patient satisfaction rates favoring ProDisc versus fusion (*P* = 0.08), which were not significant at 1-year follow-up period. Similar clinical studies and randomized trials have been conducted in the past to evaluate the performance of an artificial disc in terms of safety and efficacy [77–83].

Based on the above studies, increased facet joint loading, increased lordosis at the implanted level, hyper mobility, and wear at articulating surfaces are the major issues with TDA and need further investigations. Even though the short-term results are promising [76], the long-term complications and benefits of TDA are yet to be realized, especially in terms of preventing adjacent level disc degeneration [84, 85]. Hence, it cannot be concluded that total disc replacement is superior to spinal fusion in terms of clinical outcome, at least at present.

### 3.3. Dynamic Stabilization Systems

Spinal fusion surgeries aim at limiting the motion of the segment and restoring the stability. Anterior lumbar disc replacements are used to restore spinal alignment and kinematics of a degenerated segment. Compared to fusion of the segment, disc replacements may prevent adjacent segment degeneration. To resolve some of the deficiencies of anterior lumbar arthroplasty, such as the approach itself, difficulty of revision, and postoperative facet pain, 360° motion preservation systems based on posterior disc and posterior dynamic stabilization system (PDS) designs are being pursued [86].

Dynamic stabilization systems aim at altering favorably the movement and load transmission through the spinal motion segment [87]. The hypothesis behind dynamic stabilization system is that control of abnormal motion and more physiologic load transmission would relieve pain and prevent adjacent segment degeneration.

The biomechanical action of a dynamic stabilization system is two-fold: (i) permit or restore “normal” motion and (ii) share load with the disc and the facets. The load sharing should be more or less uniform during the entire range of motion. This implies that the kinematics of the segment stabilized with a dynamic system should be similar to the intact spine. This is achieved when the location of the instantaneous axis of rotation of the construct lies close to the intact segment [87]. There are two types of dynamic stabilizations systems currently available: dynamic pedicle screw-based systems and interspinous spacers.

#### 3.3.1. Dynamic Pedicle Screw-Based Systems

Some flexible stabilization systems consist of pedicle screws threaded into adjacent segments and a member spanning between the heads of the pedicle screws to limit the movements of the spinal segment.

In 1994 Henri Graf, (Lyon, France) introduced the Graf ligament, designed to provide less stressful load sharing. It consists of a nonelastic band as a ligament to connect the pedicle screws across the segment to be stabilized to lock the segment in full lordosis. The concept was that abnormal rotatory movement causes instability and locking the facets would control the rotation movement. The system would allow for limited flexion and no rotatory motion. The ligaments get lax in extension; hence there is no restriction in the motion [88].

The fulcrum assisted soft stabilization system (FASS system) was developed to address the disadvantages of the Graf ligament. In this system, a fulcrum is placed between the pedicle screws in front of the ligament. The fulcrum distracts the posterior annulus. When the elastic ligament is placed posterior to the fulcrum to compress the pedicle screw heads, the fulcrum transforms this posterior compression force into an anterior distraction force, which distracts the anterior annulus. The lordosis is not dependent on the patient's ability but is created by the tension in the ligament. Experimental studies have shown that the implant unloads the disc, but the flexibility of the segment is lost as greater unloading of the disc occurs by the adjustment of the tension in the ligament and the fulcrum [88].

The Dynesys system (dynamic neutralization system) was developed by Gilles and Müller. Dynesys system comprises of three components: (i) pedicle screws, (ii) polyethylene-terephthalate (PET) ligaments, and (iii) polycarbonate urethane (PCU) spacers. The spacers are bilaterally placed between the pedicle screw heads to withstand compressive loads. The ligaments are run through the hollow core of the spacers. A tensile preload of about 300 N is used to stabilize the construct [88]. The plastic cylinder between the screw heads limits the degree of lordosis that can be created. As the ligament is not elastic, flexion compresses the disc, and the axis of flexion is the posterior ligament, which is well posterior to the normal axis of flexion [88]. Active extension will open up the anterior annulus without compression of the posterior annulus. Theoretically, lordosis can be achieved by the action of the spinal extensor muscles; in extension the cylinder will take increasing load [79]. Thus, the principle of the system is its ability to create load sharing and restoration of disc height, not necessarily motion preservation because the system is rigid [87].

The Cosmic system is a pedicle screw-based dynamic instrumentation system (Ulrich, Ulm, Germany) equipped with a hinge between the screw head and threaded portion. Cosmic is a load sharing system which reduces mechanical stress on the implants. Thus, protection against implant failure and loosening is achieved. The hinged screw allows only for axial load, due to this, it is important to have a largely intact anterior column for implantation of this system. While Dynesys stabilizes by neutralizing motion, Cosmic corrects the sagittal plane and maintains motion in flexion/extension.

**Figure 9:**
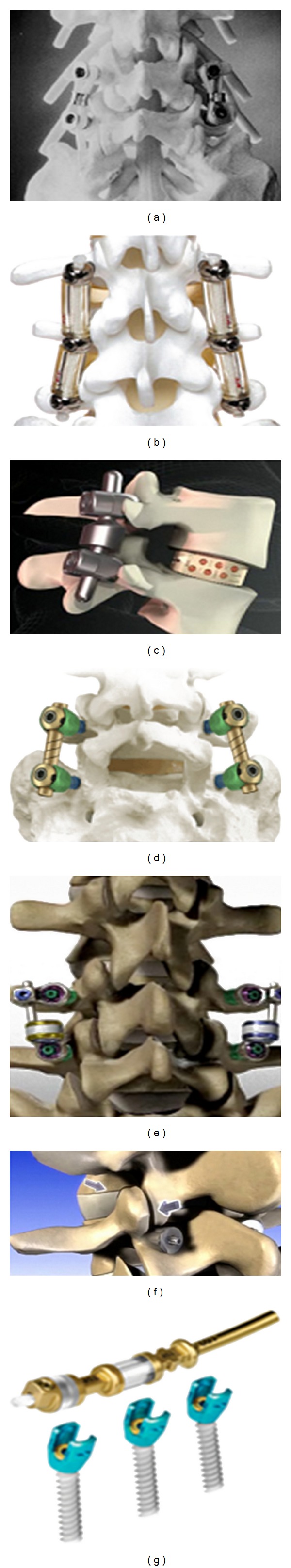
Posterior dynamic stabilization systems. (a) Graf system; (b) Dynesys; (c) IsoBar; (d) AccuFlex; (e) Stabilimax; (f) PercuDyn; (g) Transition [92].

The Isobar TTL is another novice device in this category, comprised of a titanium alloy rod and a dampener element. The dampener element is formed of a series of helical springs that allow linear and angular motion and serve as a shock absorber. This instrumentation allows flexion-extension and axial rotation, while lateral bending is restricted. A lordotic angle is also incorporated into this system. Benefits associated with this device include ease of implantation, motion segment stabilization, maintenance of lordotic angle, load sharing, and conformance to the IAR of the motion segment [89]. Other notable devices in this category include the Axient, BioFlex, TalinRod, CD Horizon Agile, and Stabilimax systems.  Figure 9 depicts some of these implants.

***In Vitro *Studies**

The Dynesys stabilization system has been widely studied. Freudiger et al. tested the Dynesys system on four cadaveric spine specimens on a lumbar spine simulator, which allowed the simultaneous application of bending moments, and compressive and shear loads. They concluded that the Dynesys reduces flexion and extension angles significantly [90].

Aylott et al. investigated the stresses of the intervertebral discs at the instrumented and the adjacent segments under compressive loading (1 kN) in flexion (6°) and extension (4°), in an *in vitro* study. The effects of spacer height on the intradiscal pressure distribution were also evaluated. They observed that Dynesys eliminated the peak stresses in the anterior annulus in flexion and in extension. The peak annulus stresses increased with decrease in the spacer height. However, there was no change in the stresses in the adjacent segment discs [91].

Niosi et al. (2004) conducted an *in vitro* biomechanical study to investigate the effect of spacer length of Dynesys on the range of motion. The test conditions included intact, injury at L3-L4, and Dynesys at L3-L4 (standard spacer, long spacer, and short spacer). They quantified range of motion and facet contact loads for a pure moment of ±7.5 Nm with and without a preload of 600 N. The trends in motion were similar with and without preload. Long spacer reduced the motion more than other two cases, the contact loads of the long and short spacer were 150% and 64% of the standard spacer, respectively [93].

Wilson et al. investigated 10 cadaveric lumbar spine specimens, subjected to pure moments of ±7.5 Nm (axial rotation, flexion, and extension) to compare range of motion and facet loads of intact specimens with those of injured specimens stabilized with Dynesys. The facet loads were measured using thin film electroresistive pressure sensors. They found that the facet loads decreased in axial rotation after implantation of Dynesys. In extension, they were similar to the intact spine, and no significant difference compared to the intact case. They, however, found that the facet loads were significantly higher in flexion with the Dynesys due to device compression. It was found that the Dynesys system reduced spinal motion from intact and decreased peak facet loading [94].

In addition to this, Schmoelz et al. [95] compared Dynesys to a rigid fixation system. They concluded that Dynesys provides substantial stability in case of degenerative pathologies and can replace conventional fusion surgery in these indications, while the motion segment is preserved.

**FE Analyses**

Rohlmann et al. studied the intersegmental rotations and intradiscal pressures in a degenerated disc after implanting the posterior dynamic implant in a FE-based study [96]. Motion at the implanted level decreased, and it slightly increased at the adjacent level. Intradiscal pressure was also decreased at the injured level with the implant. There is no much effect on IDP at the adjacent level with the implant.

In a study performed by Parepalli [97], rigid rod (fusion) system was compared with AXIENT to evaluate the parameters like range of motion, intradiscal pressure, and facet loads of the implanted and adjacent levels. They found that AXIENT restored kinematics of the degenerated spine close to normal thanthat with the fusion device (for grade I and grade II degenerated spine). AXIENT was able to restore the kinematics of degenerated spine at the adjacent levels where as fusion increased segmental motion beyond the intact. Also, stresses in pedicle screws were more for rigid system compared to the AXIENT system implicating less risk of screw breakage for AXIENT system.

Vishnubhotla et al. [98] performed a study in which FE analysis has been used to assess the kinematics of a motion segment instrumented with (i) Rigid screw rod system (used infusion), (ii) Rigid screw system with flexible rod (Nitinol; super elastic), (iii) Dynesys (Zimmer, Inc.) a pedicle screw-based dynamic stabilization system, (iv) Cosmic (Ulrich, Ulm. Germany) a pedicle screw based hinged dynamic stabilization system, and (v) Wallis (Spinal Concepts, Inc.) an interspinous based dynamic stabilization system. They reported that the dynamic stabilization systems are more flexible than rigid systems but not flexible enough to say that they preserve motion. However, the evaluation of the IAR indicates that the Dynesys system achieves kinematics closer to that of the intact spine while restricting motion.

Another study was performed by Goel et al. [99] to evaluate the biomechanical performance of the Dynesys dynamic stabilization system as a function of graded facetectomies, including complete bilateral facetectomies. An experimentally validated FE model was used to compare the biomechanics of L3-S1 lumbar spine with graded facetectomy (50%, 75%, and total bilateral medial facetectomy) at L4-L5 before and after placement of Dynesys versus intact. A 400 N compressive follower load plus a 10 Nm bending moment were applied to all models to simulate physiologically relevant motions in all planes. Results depicted the Dynesys dynamic stabilization system constrains the motion of the decompressed segment similar to a rigid system. They reported that multiple grades of facetectomy show minimal effects on the kinematics of the stabilized segment in all loading cases, except in axial rotation (AR). In total facetectomy case, increased motion and elevated pedicle screw stresses were observed in AR as compared to the intact-stabilized case. Higher screw stresses in AR for 50% facetectomies may accelerate screw loosening/failure especially in combination with other motions like flexion/extension during daily activities.

#### 3.3.2. Interspinous Spacers

The interspinous distraction devices are floating devices, which are not rigidly connected to the vertebrae. The interspinous spacers are designed to off load the posterior disc and the facet joint, by distracting the spinous processes [98]. There are several interspinous-based devices.

The Weiss springs consist of springs anchored to the lamina; the indication for the usage of this system is for fracture and deformity applications [100]. This system was modified further to consist of a rod portion attached to the spinous process using bands; these rods were meant to control rotation. A comparison study with the Harrington distraction rods concluded that modified Weiss springs often maintain better spinal stability [101].

The X-stop is intended to provide a minimally invasive, nonfusion, alternative to current treatments for degenerative lumbar spinal stenosis from L2–L5 levels, which include medical management, epidural steroid injections, and decompressive laminectomy with or without fusion. The X-stop is made of high strength titanium alloy and consists of two parts. The device is introduced between the spinous processes of adjacent level vertebral bodies and is held in place by the supraspinous ligament keeping the segment in a slightly flexed position. Due to the slightly flexed position, the nerves get decompressed thus providing relief from pain.

**Figure 10:**
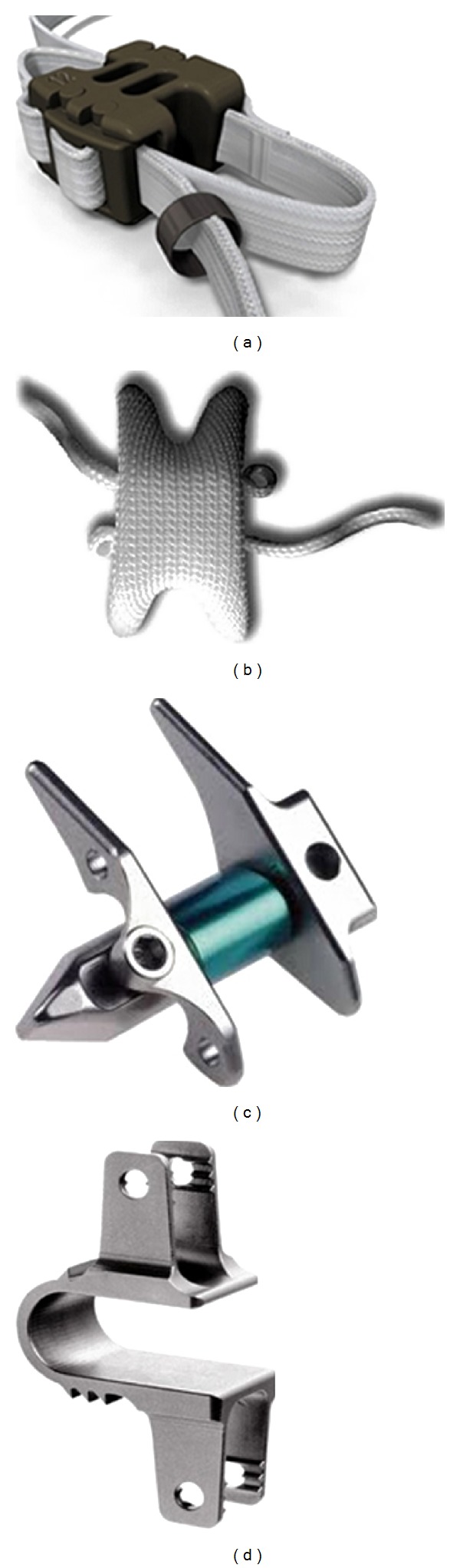
Interspinous spacers. (a) Wallis system; (b) DIAM system; (c) X-stop; (d) Coflex [92].

French orthopedic surgeon Jean Taylor developed this device. The device for intervertebral-assisted motion (DIAM) system consists of a polymeric interspinous spacer, with extended wings to act as a posterior shock-absorbing device. It consists of a flexible spacer and dual independent ligaments, which attach the spacer to the spinous process above and below, transferring some of the axial load to the posterior elements in flexion and extension (Figure 9). The flexible spacer is made with an inert medical-grade silicone core material, and the ligament is made of Graf/Senegas ligament. The surgical procedure involved for the DIAM device is to distract the spinous process to place the spacer and then to insert each ligament into the adjacent interspinous space. There is minimal wear debris seen in the DIAM, since there are no articulating surfaces. Other notable devices in this category include Coflex.  Figure 10 depicts some of these spacers.

There are few biomechanical and clinical studies showing the effectiveness of these kinds of devices. Wilke et al. did a biomechanical study on X-stop, Wallis, Coflex, and DIAM devices to assess the flexibility, stability provided, and the effect on intradiscal pressure after implanting these devices. They found that all the devices provided stability in extension, but there was no difference for flexion, lateral bending, and axial rotation. The intradiscal pressure dropped in extension and led to no difference in other mentioned loading modes [102]. 

Six human cadaveric motion segments were subjected to complex cyclic loading to determine the risk of interspinous spacer (Superion, VertiFlex Inc, CA, USA) device migration and to assess damage on the device and specimen under extreme coupled motion [103]. Motion segments with interspinous spacer were tested for 5-degree extension/10-degree flexion coupled with an axial rotation of ±3-degree up to 57600 million cycles. CT images were taken for specimens in neutral, 5-degree extension, and 10-degree flexion before and after the implantation of the spacer. Vertebral foramen and canal dimensions were quantified. Results have shown no device migration or subsidence. Specimens did not sustain any significant injury during testing. Canal area was minimally altered and foramen height, width, and area increased in extension and were statistically significant as compared to intact. It was concluded that interspinous spacer effectively prevents the motion at the implanted level and does not change the anatomy significantly.

Kabir et al. conducted a review study to find out the clinical and biomechanical evidences of interspinous device safety, effectiveness to suggest the clinical indications for these kinds of devices. They reviewed articles related to the aforementioned 4 interspinous spacers. They found that most of the studies were conducted related to X-stop, and a few studies, both biomechanical and clinical, were conducted related to other devices. In biomechanical points of view, all the devices have a beneficial effect on the kinematics of spine. The authors found these implants to be very effective in comparison to conservative treatments. They could not suggest clinical indications for interspinous devices because of varied outcomes, and a small number of studies conducted so far [63, 104]. In spite of the varied results of these interspinous devices, the author found these implants are effective in treating stenosis when compared to conservative treatment. The authors suggested the need for randomized controlled studies to evaluate these devices and to revise clinical indications for these kinds of devices [104].

## 4. Conclusion

In contrast to the previous paradigms of rigid fixation, new technologies aim to restore and preserve motion while enabling a proper load sharing. In theory, proper load sharing and restoration of physiologic motion will reduce the probability of adjacent segment disease. Current focus of research efforts emphasizes long-term evaluation of devices and validation of theoretical and experimental benefits in a clinical setting. In addition to bench-top testing, well-designed, randomized clinical trials are needed to achieve these goals.

## Figures and Tables

**Table 1:** Table lists the classification of levels of disc degeneration. Adapted from Pfirrmann et al. [28].

Grade	Structure	Distinction of nucleus and annulus	Signal intensity	Height of intervertebral disc
I	Homogeneous; bright white	Clear	Hyperintense; isointense to cerebrospinal fluid	Normal
II	Inhomogeneous with or without horizontal bands	Clear	Hyperintense; isointense to cerebrospinal fluid	Normal
III	Inhomogeneous; gray	Unclear	Intermediate	Normal to slightly decreased
IV	Inhomogeneous; gray to black	Lost	Intermediate to hypointense	Normal to moderately decreased
V	Inhomogeneous; black	Lost	Hypointense	Collapsed disc space

**Table 2:** Table lists different lumbar artificial discs and respective types of materials and features [60–63] .

Lumbar discs	Articulating surfaces and materials	Constraint	Center of rotation	Manufacturer
SB Charité	Metal-polymer-metal	Unconstrained	Mobile	DePuy Spine, Raynham, MA, USA
Prodisc-L	Metal-polymer-metal	Semiconstrained	Mobile	Synthes, West Chester, PA,USA
Maverick	Metal-metal	Semiconstrained	Fixed	Medtronic, Minneapolis, MN, USA
Flexicore	Metal-metal	Fully constrained	Fixed	Stryker, Kalamazoo, MA, USA
Mobidisc	Metal-metal	Unconstrained	Mobile	LDR medical, Troyes, France
Activ-L	Metal-polymer-metal	Semiconstrained	Mobile	Aesculap AG Tuttlingen, Germany
Kineflex	Metal-metal	Semiconstrained	Mobile	Spinal Motion, South Africa
Acroflex	Rubber core with titanium endplates (elastomeric disc)	Unconstrained	Mobile	DePuy Spine, Raynham, MA, USA

## References

[1] Modic MT, Ross JS (2007). Lumbar degenerative disk disease. *Radiology*.

[2] Crow WT, Willis DR (2009). Estimating cost of care for patients with acute low back pain: a retrospective review of patient records. *The Journal of the American Osteopathic Association*.

[3] Schizas C, Kulik G, Kosmopoulos V (2010). Disc degeneration: current surgical options. *European Cells & Materials*.

[4] Kuslich SD, Ulstrom CL, Michael CJ (1991). The tissue origin of low back pain and sciatica: a report of pain response to tissue stimulation during operations on the lumbar spine using local anesthesia. *Orthopedic Clinics of North America*.

[5] An HS, Anderson PA, Haughton VM (2004). Introduction. Disc degeneration: summary. *Spine*.

[6] Goel VK, Kim YE (1989). Effects of injury on the spinal motion segment mechanics in the axial compression mode. *Clinical Biomechanics*.

[7] Raj PP (2008). Inter vertebral disc: anatomy-physiology-patho physiology-treatment. *Pain Practice*.

[8] Schnake KJ, Putzier M, Haas NP, Kandziora F (2006). Mechanical concepts for disc regeneration. *European Spine Journal*.

[9] Adams MA, Roughley PJ (2006). What is intervertebral disc degeneration, and what causes it?. *Spine*.

[10] Urban JPG, Roberts S (2003). Degeneration of the intervertebral disc. *Arthritis Research and Therapy*.

[11] Wilke HJ, Neef P, Caimi M, Hoogland T, Claes LE (1999). New in vivo measurements of pressures in the intervertebral disc in daily life. *Spine*.

[12] Heuer F, Schmidt H, Wilke HJ (2008). The relation between intervertebral disc bulging and annular fiber associated strains for simple and complex loading. *Journal of Biomechanics*.

[13] Adams MA, McNally DS, Dolan P (1996). ’Stress’ distributions inside intervertebral discs. The effects of age and degeneration. *Journal of Bone and Joint Surgery—Series B*.

[14] Wilke HJ, Rohlmann F, Neidlinger-Wilke C, Werner K, Claes L, Kettler A (2006). Validity and interobserver agreement of a new radiographic grading system for intervertebral disc degeneration: part I. Lumbar spine. *European Spine Journal*.

[15] Schmidt H, Kettler A, Rohlmann A, Claes L, Wilke HJ (2007). The risk of disc prolapses with complex loading in different degrees of disc degeneration—a finite element analysis. *Clinical Biomechanics*.

[16] Goel VK, Weinstein JN (1990). *Biomechanics of the Spine: clinical and Surgical Perspective*.

[17] Keller HT, Holm S The load of the porcine lumbar spine during seated whole body vibration.

[18] Goel VK, Kim YE, Zhang F Biomechanical effects of vibration on the human spine.

[19] Pope MH, Kaigle AM, Magnusson M, Broman H, Hansson T (1991). Intervertebral motion during vibration. *Proceedings of the Institution of Mechanical Engineers, Part H*.

[20] Kelsey JL, Githens PB, White AA (1984). An epidemiologic study of lifting and twisting on the job and risk for acute prolapsed lumbar intervertebral disc. *Journal of Orthopaedic Research*.

[21] Kong WZ, Goel VK, Gilbertson LG, Weinstein JN (1996). Effects of muscle dysfunction on lumbar spine mechanics: a finite element study based on a two motion segments model. *Spine*.

[22] Wang S, Xia Q, Passias P, Li W, Wood K, Li G (2011). How does lumbar degenerative disc disease affect the disc deformation at the cephalic levels in vivo?. *Spine*.

[23] Li W, Wang S, Xia Q (2011). Lumbar facet joint motion in patients with degenerative disc disease at affected and adjacent levels: an in vivo biomechanical study. *Spine*.

[24] Panjabi MM, Krag MH, Chung TQ (1984). Effects of disc injury on mechanical behavior of the human spine. *Spine*.

[25] Mimura M, Panjabi MM, Oxland TR, Crisco JJ, Yamamoto I, Vasavada A (1994). Disc degeneration affects the multidirectional flexibility of the lumbar spine. *Spine*.

[26] McNally DS, Adams MA (1992). Internal intervertebral disc mechanics as revealed by stress profilometry. *Spine*.

[27] Shirazi-Adl A, Ahmed AM, Shrivastava SC (1986). A finite element study of a lumbar motion segment subjected to pure sagittal plane moments. *Journal of Biomechanics*.

[28] Pfirrmann CWA, Metzdorf A, Zanetti M, Hodler J, Boos N (2001). Magnetic resonance classification of lumbar intervertebral disc degeneration. *Spine*.

[29] Goel VK, Pope MH (1995). Biomechanics of fusion and stabilization. *Spine*.

[30] Wilke HJ, Wenger K, Claes L (1998). Testing criteria for spinal implants: recommendations for the standardization of in vitro stability testing of spinal implants. *European Spine Journal*.

[31] Goel VK, Thomas NA, Charles CR, Weinstein NK, James N (1987). A technique to evaluate an internal spinal device by use of the selspot system: an application to luque closed loop. *Spine*.

[32] Goel VK, Wilder DG, Pope MH, Edwards WT, McLain RF, Boden SD (1995). Biomechanical testing of the spine: load-controlled versus displacement- controlled analysis. *Spine*.

[33] Goel VK, Grauer JN, Patel TC (2005). Effects of Charité artificial disc on the implanted and adjacent spinal segments mechanics using a hybrid testing protocol. *Spine*.

[34] Grauer JN, Biyani A, Faizan A (2006). Biomechanics of two-level Charité artificial disc placement in comparison to fusion plus single-level disc placement combination. *Spine Journal*.

[35] Abdullah KG, Steinmetz MP, Benzel EC, Mroz TE (2011). The state of lumbar fusion extenders. *Spine*.

[36] Wang JC, Mummaneni PV, Haid RW (2005). Current treatment strategies for the painful lumbar motion segment: posterolateral fusion versus interbody fusion. *Spine*.

[37] Bhatia NN, Lee KH, Bui CNH, Luna M, Wahba GM, Lee TQ (2011). Biomechanical evaluation of an expandable cage in single segment posterior lumbar interbody fusion. *Spine*.

[38] Wang JC, Haid RW, Miller JS, Robinson JC (2006). Comparison of CD HORIZON SPIRE spinous process plate system stabilization and pedicle screw fixation after anterior lumbar interbodyfusion: invited submission from the Joint Section Meeting on Disorders of the Spine and Peripheral Nerves. *Journal of Neurosurgery. Spine*.

[39] Wu JC, Mummaneni PV (2010). Using lumbar interspinous anchor with transforaminal lumbar interbody fixation. *World Neurosurgery*.

[40] Karahalios DG, Kaibara T, Porter RW (2010). Biomechanics of a lumbar interspinous anchor with anterior lumbar interbody fusion. *Journal of Neurosurgery. Spine*.

[41] Youssef JA, McAfee PC, Patty CA (2010). Minimally invasive surgery: lateral approach interbody fusion: results and review. *Spine*.

[42] Djurasovic M, Glassman SD, Dimar JR, Howard JM, Bratcher KR, Carreon LY (2011). Does fusion status correlate with patient outcomes in lumbar spinal fusion?. *Spine*.

[43] Kiapour A, Kiapour AM, Kodigudla MK, Mishra S, Goel VK (2012). *Biomechanics of a Novel Expandable Interbody Cage Design*.

[44] Faizan A, Kiapour A, Kiapour AM, Goel VK (2012). *Effect of Interbody Cage’s Footprint on the Endplate Stresses Immediatelyafter TLIF Surgery: A Finite Element Based Study*.

[45] Oxland TR, Hoffer Z, Nydegger T, Rathonyi GC, Nolte LP (2000). A comparative biomechanical investigation of anterior lumbar interbody cages: central and bilateral approaches. *Journal of Bone and Joint Surgery—Series A*.

[46] Rathonyi GC, Oxland TR, Gerich U, Grassmann S, Nolte LP (1998). The role of supplemental translaminar screws in anterior lumbar interbody fixation: a biomechanical study. *European Spine Journal*.

[47] Tsantrizos A, Baramki HG, Zeidman S, Steffen T (2000). Segmental stability and compressive strength of posterior lumbar interbody fusion implants. *Spine*.

[48] Kiapour A, Goel VK, Ferrara L, Hunt J (2011). *Subsidence Evaluation of 4-WEB, a Novel Cross Strut Based, Interbody Cage Design*.

[49] Kanayama M, Cunningham BW, Haggerty CJ, Abumi K, Kaneda K, McAfee PC (2000). In vitro biomechanical investigation of the stability and stress-shielding effect of lumbar interbody fusion devices. *Journal of Neurosurgery*.

[50] Kiapour A, O’Donnell J, Goel VK, Biyani A (2010). *Comparison of Biomechanics of Lumbar-Pelvis Segment with Posterior Screw-Rod Versus Interspinous Plate Fixation System*.

[51] Cakir B, Carazzo C, Schmidt R, Mattes T, Reichel H, Käfer W (2009). Adjacent segment mobility after rigid and semirigid instrumentation of the lumbar spine. *Spine*.

[52] Panjabi M, Henderson G, Abjornson C, Yue J (2007). Multidirectional testing of one- and two-level ProDisc-L versus simulated fusions. *Spine*.

[53] Panjabi M, Malcolmson G, Teng E, Tominaga Y, Henderson G, Serhan H (2007). Hybrid testing of lumbar CHARITI discs versus fusions. *Spine*.

[54] Chow DHK, Luk KDK, Evans JH, Leong JCY (1996). Effects of short anterior lumbar interbody fusion on biomechanics of neighboring unfused segments. *Spine*.

[55] Esses SI, Doherty BJ, Crawford MJ, Dreyzin V (1996). Kinematic evaluation of lumbar fusion techniques. *Spine*.

[56] Ha KY, Schendel MJ, Lewis JL, Ogilvie JW (1993). Effect of immobilization and configuration on lumbar adjacent-segment biomechanics. *Journal of Spinal Disorders*.

[57] Videbaek TS, Egund N, Christensen FB, Grethe Jurik A, Bünger CE (2010). Adjacent segment degeneration after lumbar spinal fusion: the impact of anterior column support: a randomized clinical trial with an eight-to thirteen-year magnetic resonance imaging follow-up. *Spine*.

[58] Fernstrom U (1966). Arthroplasty with intercorporal endoprosthesis in herniated disc and in painful disc. *Acta Chirurgica Scandinavica. Supplementum*.

[59] Zippel H, Schellnack K, Buttner K (1986). Exchanging intervertebral disks. The concept and clinical experience using a cement-free intervertebral disk endoprosthesis of the “Charite Modular SB” type. *Chirurgia Narzadow Ruchu i Ortopedia Polska*.

[60] Kiapour A (2010). *Investigation into lumbar spine biomechanics of 360 motion preservation systems*.

[61] Cunningham BW, Lowery GL, Serhan HA (2002). Total disc replacement arthroplasty using the Acroflex lumbar disc: a non-human primate model. *European Spine Journal*.

[62] Ha SK, Kim SH, Kim DH, Park JY, Lim DJ, Lee SK (2009). Biomechanical study of lumbar spinal arthroplasty with a semi-constrained artificial disc (Activ L) in the human cadaveric spine. *Journal of Korean Neurosurgical Society*.

[63] Anderson PA, Rouleau JP (2004). Intervertebral disc arthroplasty. *Spine*.

[64] Hitchon PW, Eichholz K, Barry C (2005). Biomechanical studies of an artificial disc implant in the human cadaveric spine. *Journal of Neurosurgery. Spine*.

[65] Rousseau MA, Bradford DS, Bertagnoli R, Hu SS, Lotz JC (2006). Disc arthroplasty design influences intervertebral kinematics and facet forces. *Spine Journal*.

[66] Goel VK, Grauer JN, Patel TC (2005). Effects of Charité artificial disc on the implanted and adjacent spinal segments mechanics using a hybrid testing protocol. *Spine*.

[67] Serhan HA, Dooris AP, Parsons ML, Ares PJ, Gabriel SM (2006). In vitro wear assessment of the charité artificial disc according to ASTM recommendations. *Spine*.

[68] Paré PE, Chan FW, Powell ML (2007). Wear characterization of the A-MAV*™* anterior motion replacement using a spine wear simulator. *Wear*.

[69] Rohlmann A, Zander T, Bergmann G (2005). Effect of total disc replacement with ProDisc on intersegmental rotation of the lumbar spine. *Spine*.

[70] Moumene M, Geisler FH (2007). Comparison of biomechanical function at ideal and varied surgical placement for two lumbar artificial disc implant designs: mobile-core versus fixed-core. *Spine*.

[71] Dooris AP, Goel VK, Grosland NM, Gilbertson LG, Wilder DG (2001). Load-sharing between anterior and posterior elements in a lumbar motion segment implanted with an artificial disc. *Spine*.

[72] Denozière G, Ku DN (2006). Biomechanical comparison between fusion of two vertebrae and implantation of an artificialintervertebral disc. *Journal of Biomechanics*.

[73] Rawlinson JJ, Punga KP, Gunsallus KL, Bartel DL, Wright TM (2007). Wear simulation of the ProDisc-L disc replacement using adaptive finite element analysis. *Journal of Neurosurgery. Spine*.

[74] Bhattacharya S, Goel VK, Liu X, Kiapour A, Serhan HA (2011). Models that incorporate spinal structures predict better wear performance of cervical artificial discs. *Spine Journal*.

[75] Siepe CJ, Korge A, Grochulla F, Mehren C, Mayer HM (2008). Analysis of post-operative pain patterns following total lumbar disc replacement: results from fluoroscopically guided spine infiltrations. *European Spine Journal*.

[76] Zigler JE (2004). Lumbar spine arthroplasty using the ProDisc II. *Spine Journal*.

[77] Tournier C, Aunoble S, Le Huec JC (2007). Total disc arthroplasty: consequences for sagittal balance and lumbar spine movement. *European Spine Journal*.

[78] Yaszay B, Bendo JA, Goldstein JA, Quirno M, Spivak JM, Errico TJ (2008). Effect of intervertebral disc height on postoperative motion and outcomes after ProDisc-L lumbar disc replacement. *Spine*.

[79] Stieber JR, Donald GD (2006). Early failure of lumbar disc replacement: case report and review of the literature. *Journal of Spinal Disorders and Techniques*.

[80] Tropiano P, Huang RC, Girardi FP, Cammisa FP, Marnay T (2005). Lumbar total disc replacement: seven to eleven-year follow-up. *Journal of Bone and Joint Surgery—Series A*.

[81] Tropiano P, Huang RC, Girardi FP, Cammisa FP, Marnay T (2006). Lumbar total disc replacement. Surgical technique. *Journal of bone and Joint Surgery—Series A*.

[82] Zigler JE, Burd TA, Vialle EN, Sachs BL, Rashbaum RF, Ohnmeiss DD (2003). Lumbar spine arthroplasty early results using the ProDisc II: a prospective randomized trial of arthroplasty versus fusion. *Journal of Spinal Disorders and Techniques*.

[83] Sasso RC, Foulk DM, Hahn M (2008). Prospective, randomized trial of metal-on-metal artificial lumbar disc replacement: initial results for treatment of discogenic pain. *Spine*.

[84] Freeman BJC, Davenport J (2006). Total disc replacement in the lumbar spine: a systematic review of the literature. *European Spine Journal*.

[85] Resnick DK, Watters WC (2007). Lumbar disc arthroplasty: a critical review. *Clinical Neurosurgery*.

[86] Goel VK, Kiapour A, Faizan A, Krishna M, Friesem T (2007). Finite Element Study of Matched Paired Posterior Disc Implant and Dynamic Stabilizer (360° Motion Preservation System). *SAS Journal*.

[87] Sengupta DK (2004). Dynamic stabilization devices in the treatment of low back pain. *Orthopedic Clinics of North America*.

[88] Mulholland RC, Sengupta DK (2002). Rationale, principles and experimental evaluation of the concept of soft stabilization. *European Spine Journal*.

[89] Kim D, Cammisa F, Kim DH (2006). *Dynamic Reconstruction of the Spine*.

[90] Freudiger S, Dubois G, Lorrain M (1999). Dynamic neutralisation of the lumbar spine confirmed on a new lumbar spine simulator in vitro. *Archives of Orthopaedic and Trauma Surgery*.

[91] Aylott CEW, McKinlay KJ, Freeman BJC, Shepperd J, McNally DS (2004). *In-Vitro Biomechanical Effects of Dynesys. Conference Abstract*.

[92] Serhan H, Mhatre D, Defossez H, Bono CM (2011). Motion-preserving technologies for degenerative lumbar spine: the past, present, and future horizons. *SAS Journal*.

[93] Niosi C, Zhu Q, Wilson DC, Keynan O, Wilson DR, Oxland T (2004). *Does Spacer Length of Dynamic Posterior Stabilization System have an Effect on Range of Motion? Conference Abstract*.

[94] Wilson DC, Niosi C, Zhu Q (2004). *How Does Loading in the Facet Joint a Change with Implantation of a Dynamic Posterior Stabilization System? Conference Abstract*.

[95] Schmoelz W, Huber JF, Nydegger T, Dipl-Ing, Claes L, Wilke HJ (2003). Dynamic stabilization of the lumbar spine and its effects on adjacent segments: an in vitro experiment. *Journal of Spinal Disorders and Techniques*.

[96] Rohlmann A, Burra NK, Zander T, Bergmann G (2007). Comparison of the effects of bilateral posterior dynamic and rigid fixation devices on the loads in the lumbar spine: a finite element analysis. *European Spine Journal*.

[97] Parepalli BK (2009). *Biomechanical Evaluation of Posterior Dynamic Stabilization Systems in Lumbar Spine,*.

[98] Vishnubhotla S, Goel V, Walkenhorst J, Boyd L, Vadapalli S, Shaw M (2005). *Biomechanical Advantages of Using Dynamic Stabilization Over Rigid Stabilization. Poster Presentation*.

[99] Kiapour A, Ambati D, Hoy RW, Goel VK (2012). Effect of graded facetectomy on biomechanics of dynesys dynamic stabilization system. *SPINE*.

[100] Benzel EC, Larson SJ (1986). Functional recovery after decompressive operation for thoracic and lumbar spine fractures. *Neurosurgery*.

[101] Maiman DJ, Sances A, Larson SJ (1985). Comparison of the failure biomechanics of spinal fixation devices. *Neurosurgery*.

[102] Wilke HJ, Drumm J, Häussler K, Mack C, Steudel WI, Kettler A (2008). Biomechanical effect of different lumbar interspinous implants on flexibility and intradiscal pressure. *European Spine Journal*.

[103] Goyal A, Goel VK, Mehta A, Dick D, Chinthakunta SR, Ferrara L (2008). Cyclic loads do not compromise functionality of the interspinous spacer or cause damage to the spinal segment: an in vitro analysis. *Journal of Long-Term Effects of Medical Implants*.

[104] Kabir SM, Gupta SR, Casey AT (2010). Lumbar interspinous spacers: a systematic review of clinical and biomechanical evidence. *Spine*.

